# Synergisms between microbial pathogens in plant disease complexes: a growing trend

**DOI:** 10.3389/fpls.2015.00385

**Published:** 2015-05-27

**Authors:** Jay Ram Lamichhane, Vittorio Venturi

**Affiliations:** ^1^INRA, UAR 1240 Eco-InnovThiverval-Grignon, France; ^2^International Centre for Genetic Engineering and BiotechnologyTrieste, Italy

**Keywords:** microbial cooperation, co-infection, synergism, interspecies interactions, disease complex

## Abstract

Plant diseases are often thought to be caused by one species or even by a specific strain. Microbes in nature, however, mostly occur as part of complex communities and this has been noted since the time of van Leeuwenhoek. Interestingly, most laboratory studies focus on single microbial strains grown in pure culture; we were therefore unaware of possible interspecies and/or inter-kingdom interactions of pathogenic microbes in the wild. In human and animal infections, it is now being recognized that many diseases are the result of multispecies synergistic interactions. This increases the complexity of the disease and has to be taken into consideration in the development of more effective control measures. On the other hand, there are only a few reports of synergistic pathogen–pathogen interactions in plant diseases and the mechanisms of interactions are currently unknown. Here we review some of these reports of synergism between different plant pathogens and their possible implications in crop health. Finally, we briefly highlight the recent technological advances in diagnostics as these are beginning to provide important insights into the microbial communities associated with complex plant diseases. These examples of synergistic interactions of plant pathogens that lead to disease complexes might prove to be more common than expected and understanding the underlying mechanisms might have important implications in plant disease epidemiology and management.

## Introduction

A widely accepted current concept is that a pathogen colonizes a host and responds to the host environment resulting in the manipulation of expression of its resistance genes. Many studies have demonstrated this type of host–pathogen interaction in monospecies infections. In contrast to this, in the wild, microbes have been known to be part of complex multispecies consortia/communities since the time of van Leeuwenhoek during 1600s (Gest, 2004). The earliest reports regarding microbial communities as causal agents of a disease are attributed to Pasteur in the 1800s who observed that a disease can also be due to synergistic interactions of different microorganisms. Although microbial plant diseases of definite etiology are still mainly considered as being caused by single microbial cultures, evidence is now beginning to mount that there can be synergisms between different pathogens in complex plant diseases.

In human pathology, there is now a growing awareness that infectious agents frequently do not operate alone and their virulence can be affected by their interaction with other commensals or pathogens (Singer, 2010). Consequently, the study of multispecies synergistic interactions is emerging as a new important subject for better understanding of microbial diseases (Short et al., 2014). Several examples of synergistic commensal–pathogen and pathogen–pathogen interactions that lead to increased human disease severity have been recently reported (Singer, 2010; Peters et al., 2012; Bosch et al., 2013). Similar examples of pathogen–pathogen synergistic interactions that result in increased disease severity in animals have also been reported (Harms et al., 2001; Ellis et al., 2004). For example, polyparasitism as a decisive factor has been recently demonstrated in a protozoal disease in marine mammals; more precisely, co-infections of *Toxoplasma gondii* and *Sarcocystis neurona* were more recurrently related with mortality and protozoal encephalitis when compared to solitary infections (Gibson et al., 2011).

In contrast to mammalian pathology, the concept of monospecies/monostrain infections is more deeply rooted in plant pathology. In such cases, plant disease epidemics have almost exclusively been associated to a single pathogen belonging to a clonal group. Examples are bacterial canker of kiwifruit (Mazzaglia et al., 2012), bleeding canker of horse chestnut (Green et al., 2010), diseases of fruit and nut trees (Hajri et al., 2012), Bayoud disease of date palm (Tantaoui et al., 1996) and potato late blight (Goodwin et al., 1995). None of these studies have focused on the possible role of other microbial populations associated with infected plants in disease outcome. This is not surprising as new methodologies to analyze total microbial populations from diseased plant tissues are only beginning to be available on a routine basis. The few studies, using traditional approaches, reveal that many plant species can frequently be infected at the same time by more than one pathogenic species (Fitt et al., 2006); in many cases, a single microbe infection may not result in severe disease symptoms while the co-infection with another microbial species may lead to severe disease development due to synergistic interactions.

The objective of this review is to highlight, describe, and discuss known examples of plant diseases which involve pathogen–pathogen interactions. There are two main possibilities in which different plant pathogens interact: (i) a disease complex where disease is the result of the interaction of several plant pathogens belonging to the same species or phylum, (ii) a disease complex due to different plant pathogens belonging to different phyla. This review does not cover plant diseases involving commensal–pathogen, nematode–microbe, or insect–microbe interactions. The universality of these synergistic interactions between plant pathogens in the living world is an alarm bell and microbiologists and plant pathologists need therefore to better consider in the future that one pathogenic agent can team up with others rather than acting alone.

## Synergistic Pathogen–Pathogen Interactions

Plant diseases where more than one pathogen is involved in the infection process are commonly termed as “complex” since their diagnosis and subsequent control are more complicated. Such diseases occur as a result of a network that involves a wide range of microbial interactions. Monoculture inoculations are commonly performed to evaluate the pathogenicity behavior of a given pathogen. Consequently, our knowledge of their possible synergism that leads to increased disease severity is poor. It is likely that synergism among different pathogens leading to more severe disease symptoms occur more often than expected (Begon et al., 2006). Such synergistic interactions in plants may be of crucial importance for the understanding of microbial pathogenesis and evolution and consequent development of effective disease control strategies.

A non-exhaustive list of synergistic pathogen–pathogen infections in plants that often lead to increased disease severity is reported in **Table 1**. This overall picture is beginning to facilitate the understanding of epidemiology and control of numerous complex diseases. There are now several recent reports on the diagnosis of complex diseases and their successful management (Gleason et al., 2011; Clark et al., 2012; Martin et al., 2013; Freeman et al., 2014).

**Table 1:** Pathogen–pathogen synergistic interactions that lead to plant disease occurrence and increased disease severity.

Host	Disease	Causal agents	Reference
**Bacteria–bacteria**		
Tomato	Pith necrosis	*Pseudomonas corrugata* and *P. mediterranea*	Moura et al. (2005)
		*P. corrugata* and *P. marginalis*	Kůdela et al. (2010)
		*P. corrugata, P. Mediterranea,* and *P. marginalis*	Moura et al. (2005), Kůdela et al. (2010)
Mulberry	Wilt	*Enterobacter asburiae* and *Enterobacter* sp.	Wang et al. (2010)
Sugarbeet	Leaf spot	*Xanthomonas* sp.	Mbega et al. (2012)
Broccoli	Head rot	*P. marginalis, Erwinia carotovora, P. fluorescens,* and *P. viridiflava*	Canaday et al. (1991)
Potato	Zebra complex	*Candidatus liberibacter solanacearum* and *Candidatus liberibacter psyllaurous*	Wen et al. (2009)
**Fungi–fungi**		
*Acacia mangium*	Root rot	*Ganoderma philippii, G. mastoporum, G. aff. steyaertanum, G. austral,* and *Amauroderma rugosum*	Glen et al. (2009)
Apple	Sooty blotch and flyspeck	*Zygophialasp, Microcyclospora,* and *Microcyclosporella*	Batzer et al. (2008), Frank et al. (2010)
Banana	Sigatoka	*Mycosphaerella fijiensis, M. musicola,* and *M. eumusae*	Arzanlou et al. (2007)
Cassava	Root rot	*Fusarium sp. Botryodiplodia theobromae* and *Armillaria* sp.	Bandyopadhyay et al. (2006)
Cereals	Bare patch disease	*Rhizoctonia* sp.	Roberts and Sivasithamparam (1986)
	*Fusarium* head blight	*Fusarium graminearum species complex*	Del Ponte et al. (2014)
Chestnut	Ink disease	*Phytophtora* sp.	Vettraino et al. (2005)
Coffee	Anthracnose	*Colletotrichum* sp.	Nguyen et al. (2010)
Cranberry	Fruit rot	A large number of species from different genera	Oudemans et al. (1998), Olatinwo et al. (2003)
Cymbidium	Yellow leaf spot	*F. subglutinans* and *F. proliferatum*	Ichikawa and Aoki (2000)
Eucalyptus	Leaf spot	*Teratosphaeria juvenalis* and *T. verrucosa*	Crous et al. (2009)
Hazelnut	Gray necrosis	*Alternaria* sp., *Fusarium* sp., and *Phomopsis* sp.	Belisario et al. (2004)
Grapevines	Grapevine decline	*Botryosphaeriaceae* sp. and *Ilyonectria* sp.	Whitelaw-Weckert et al. (2013)
	Black dead arm	*Botryosphaeria dothidea, Diplodiaseriata,* and *Lasiodiplodia theobromae*	Larignon et al. (2001), van Niekerk et al. (2006)
	Black foot	*Cylindrocarpon liriodenderi* and *C. macrodidymum*	Alaniz et al. (2007)
	Petri and esca	*Phaeomoniella chlamydospora* and *Phaeoacremonium aleophilum*	Edwards and Pascoe (2004)
	Anthracnose	*Elsinoeampelina, Colletotrichum gloeosporioides,* and *C. acutatum*	Sawant et al. (2012)
Leek	Leaf blotch	*A. porri* and *Stemphylium vesicarium*	Suheri and Price (2001)
Mango	Malformation	*F. mangiferae, F sterilihyphosum, F. mexicanum, F. tupiense, F. proliferatum,* and *F. pseudocircinatum*	Freeman et al. (2014)
Maize	Ear rot	*F. meridionale* and *F. boothii*	Sampietro et al. (2011)
	Root and stalk rot	*Trichoderma* sp., *Penicillium* sp., *Pyrenochaeta indica, F. moniliforme, F. graminearum,* and *F. oxysporum*	Ramsey (1990)
	Crown and root rot	*F. boothii, F. graminearum,* and *F. meridionale*	Lamprecht et al. (2011)
Milkwort	Decline	*F. oxysporum* and *F. solani*	Vitullo et al. (2014)
Millet	Stalk rot	*Bipolarissetariae, F. semitectum,* and *F. moniliforme*	Wilson (2002)
Oilseed rape	Phoma canker	*Leptosphaeria biglobosa* and *L. maculans*	Fitt et al. (2006)
Pea	*Ascochyta* blight	*Mycosphaerella pinodes, Phoma medicaginis* var. *pinodella,* and *P. glomerata*	Le May et al. (2009)
		*P. medicaginis* var. *pinodella* and *F. oxysporum* f. sp. *Pisi*	Sagar and Sugha (1997)
Periwinkle	Anthracnose	*Colletotrichum siamense* and *C. tropicale*	Tomioka et al. (2013)
Potato	Leaf spots	*A. tenuissima, A. dumosa, A. arborescens, A. Infectoria,* and *A. interrupta*	Ardestani et al. (2010)
Rice	Bakanae disease	*F. fujikuroi* and *F. proliferatum*	Voigt et al. (1995)
Soybean	Wilt	*F. graminearum, F. Meridionale,* and *F. cortaderiae*	Barros et al. (2014)
Stone fruit	Brown rot	*Monilinialaxa* and *M. fructicola*	Papavasileiou et al. (2014)
Strawberry	Root rot	*Pythium* sp., *Fusarium* sp., *Cylindrocarpon* sp., *Rhizoctonia* sp.	Wing et al. (1994)
Wheat	Septoria leaf Blotches	*Septoriatritici* and *Stagonosporanodorum*	Fitt et al. (2006)
	Stem eyespot	*Oculimaculayallundae* and *O. acuformis*	Fitt et al. (2006)
	Foot and crown rot	*F. graminearum, F. culmorum, F. poae,* and *F. sporotrichioides*	Kuzdraliński et al. (2014)
Apple	Replant disease	*Cylindrocarpon destructans, Phytophthora cactorum,* and *Pythium* sp.	Mazzola (1998)
Ginger	Soft rot	*Pythium* sp.	Le et al. (2014)
Parsnip and parsley	Root rot	A large number of *Pythium* sp.	Petkowski et al. (2013)
**Virus–virus**			
Corn	Corn lethal necrosis	*Maize chlorotic mottle virus* (MCMV) and *Wheat streak mosaic virus* (WSMV)	Niblett and Claflin (1978), Scheets (1998)
Cowpea	Cowpea stunt	*Cucumber mosaic virus* (CMV) and black-eye *Cowpea mosaic virus* (BCMV)	Pio-Ribeiro et al. (1978)
Pepper	Not assigned	CMV and *Pepper mottle virus*	Murphy and Bowen (2006)
Soybean	Not assigned	*Soybean mosaic virus* and *Bean pod mottle virus*	Calvert and Ghabrial (1983)
Sweet potato	Not assigned	*Sweet potato feathery mottle virus* and *Sweet potato chlorotic stunt virus*	Karyeija et al. (2000)
Sweet potato	Not assigned	*Begomoviruses* with *Sweet potato chlorotic stunt virus*	Cuellar et al. (2015)
Tobacco	Not assigned	*Potato virus* X and Y	Damirdagh and Ross (1967)
Wheat	Not assigned	WSMV and *Triticum mosaic virus*	Tatineni et al. (2010)
Zucchini squash	Not assigned	CMV and *Zucchini yellow mosaic virus*	Choi et al. (2002), Wang et al. (2002)
Blackberry	Blackberry yellow vein disease (BYVD)	*Tobacco ringspot virus, Raspberry bush dwarf virus* and *Crinivirus*	Martin et al. (2013)
Raspberry	Raspberry mosaic disease	*Raspberry necrosis virus, Raspberry leaf mottle virus,* and *Rubus yellow net virus*	Martin et al. (2013)
Raspberry	Crumbly fruit complex	*Raspberry bushy virus* and *Raspberry latent virus*	Martin et al. (2013)
Carrot	Motley dwarf	*Carrot red leaf luteovirus* and *Carrot mottle umbravirus*	Watson et al. (1998)
Cucurbits	mosaic disease	*Watermelon mosaic virus* and *Zucchini mosaic virus*	Salvaudon et al. (2013)
Sweet potato	Viral decline	*Sweet potato chlorotic stunt virus* and *Sweet potato feathery mottle virus*	Clark et al. (2012)
		*Sweet potato virus Y and Sweet potato feathery mottle virus*	Tairo et al. (2006), Gibson and Kreuze (2014)
Turfgrass	Decline	*Panicum mosaic virus* and *Satellite panicum mosaic virus*	Cabrera and Scholthof (1999)
Grapevine	Vein-clearing	*Phaeoacremonium aleophilum* and *Grapevine rupestris stem pitting-associated virus*	Lunden et al. (2010)
	Leaf roll	*Grapevine leafroll-associated viruses*	Naidu et al. (2014)
**Mixed infections**			
Potato	Potato early dying	*Verticillium dahlia* and *Pectobacterium* sp.	Dung et al. (2013)
Arrowleaf Clover	Root disease	*Pythiumsp, Rhizoctonia* sp., and *Fusarium* sp.	Pemberton et al. (1998)
Sugar beet	Root rot	*Leuconostocmesenteroides* subsp. *dextranicum, Lactobacillus, Gluconobacter, Rhizoctonia*	Strausbaugh and Gillen (2008), Strausbaugh and Eujayl (2012)
Walnut	Brown apical necrosis	*Fusarium, Alternaria, Cladosporium, Colletotrichum, Phomopsis,* and *Xanthomonas arboricola*	Belisario et al. (2002)
Pumpkin	Gummy stem blight and black rot	*Didymella bryoniae, Pectobacterium carotovorum, Pseudomonas viridiflava, P. syringae*, and *X. cucurbitae*	Grube et al. (2011)
*Panax notoginseng*	Root rot	*Alternaria* sp., *Cylindrocarpon* sp., *Fusarium* sp., *Phytophthora cactorum, Phoma herbarum, Rhizoctonia solani, Pseudomonas* sp., *and Ralstonia* sp.	Miao et al. (2006), Ma et al. (2013)

### Bacteria–Bacteria Interactions

Tomato pith necrosis is thus far a leading example of co-infection due to synergistic interactions among several bacterial pathogens. Overall, eight bacterial species namely *Pseudomonas cichorii* (Wilkie and Dye, 1974), *P. corrugata* (Scarlett et al., 1978), *P. viridiflava* (Goumas and Chatzaki, 1998), *P. mediterranea* (Saygili et al., 2008), *P. fluorescens* (Saygili et al., 2008), *Pectobacterium atrosepticum* (Malathrakis and Goumas, 1987), *Pectobacterium carotovorum* (Dhanvanthari and Dirks, 1987; formerly *Erwinia*), and *Dickeya chrysanthemi* (Formerly *Erwinia*; Alivizatos, 1985) can cause tomato pith necrosis alone or in association with the other bacterial species. The severity of the disease is greatly enhanced when co-infection of one or more bacterial species occurs. In particular, co-infection of *P. corrugata*–*P. marginalis,* or *P. corrugata*–*P. mediterranea* have been reported to cause severe infection in tomato (Moura et al., 2005; Saygili et al., 2008; Kůdela et al., 2010). Similarly, bacterial soft or head rot of broccoli is another complex disease caused by numerous plant pathogenic bacteria. Overall, *Pectobacterium carotovorum, P. marginalis*, *P. fluorescens,* and *P. viridiflava* have been reported to cause broccoli head rot (Canaday et al., 1991). Bacterial strains belonging to these species are also capable of causing soft rot on unwounded broccoli when co-inoculated. The mechanism(s) for this cooperativity among different bacterial species is currently unknown.

### Fungi–Fungi Interactions

Synergistic interactions between different fungal pathogens have been studied intensively. For example, the young grapevine decline disease is present across many regions worldwide, and is caused by the following fungal pathogens when present alone: *Ilyonectris* sp., *Phaeomoniella chlamydospora*, *Togninia* sp., and *Botryosphaeriaceae* sp. (Mugnai et al., 1999; Gramaje and Armengol, 2011). The fungal causal agent(s) vary considerably between the grapevine producing regions. A recent study demonstrated that co-infection of several fungal species belonging to *Botryosphaeriaceae* sp. and *Ilyonectria* sp. results in very severe decline of young grafted grapevines in the field (Whitelaw-Weckert et al., 2013). Similarly, laboratory experiments further confirmed that co-inoculation of *Ilyonectria* and *Botryosphaeriaceae* isolates led to an increased disease severity compared to monoculture inoculations of *Ilyonectria* isolates (Whitelaw-Weckert et al., 2013). The different pathogens isolated from grapevine decline symptoms throughout cultivated areas are likely to have co-evolved due to their close association. Prior to these studies, only one pathogen was thought to be the causal agent of grapevine decline depending on the region due to a marked dominance of one species over all the other ones for each infection.

Numerous examples of co-existence of fungal pathogens on arable crops has been described in the UK (Fitt et al., 2006). Such examples include a disease complex of wheat leaves known as septoria leaf blotches caused by *Septoria tritici* and *Stagonospora nodorum*; wheat stem affected by *Oculimacula yallundae* and *O. acuformis* and phoma stem canker on oilseed rape caused by *Leptosphaeria biglobosa* and *L. maculans*. Another complex disease of wheat caused by a group of *Fusarium* species is foot and crown rot. Overall, four species of the pathogen (*Fusarium graminearum, F. culmorum, F. poae, and F. sporotrichioides*) are associated with the disease although their prevalence differs from one geographic region to another (Kuzdraliński et al., 2014). It has also been reported that the majority of fields in eastern Poland are subjected to the attack of at least one or two of *Fusarium* species. The presence of *F. graminearum* was found to foster the occurrence of *F. culmorum* and this result was observable also for *F. poae* and *F. sporotrichioides*. An additional disease complex of major economic significance worldwide is *Fusarium* head blight, especially due to the grain contamination with harmful mycotoxins produced by the fungus during pathogenesis (McMullen et al., 2012). Over 16 known species of the *F. graminearum* species complex have been reported as the causal agent of *Fusarium* head blight (O’Donnell et al., 2008; Yli-Mattila et al., 2009; Sarver et al., 2011). Studies carried out in Brazil show that the prevalence of the species in *Fusarium* head blight varies from one geographic region to another (Del Ponte et al., 2014). Our current knowledge is very poor concerning mechanisms that explain the geographic variation and prevalence of specific pathogens in plants affected by a particular complex disease and it is possible that such variations are related to the ecological preference of these pathogens. Moreover, abiotic factors and cultural practices might also influence this variation in pathogen prevalence.

Black spot disease complex of pea was previously known to be caused by three fungal pathogens (Le May et al., 2009); however, four additional pathogens have been recently found in association with this disease. These new pathogens include *Phoma koolunga* (Davidson et al., 2009), *Phoma herbarum* (Li et al., 2011), *Boerema exigua* var. *exigua* (Li et al., 2012), and *Phoma glomerata* (Tran et al., 2014). All these fungi are necrotrophic, generalist and polyphagous species and these characteristics favor colonization of new environments. It is possible that these pathogens use synergism as a strategy to infect a large variety of plants which might also explain why some pathogens occur more easily than others in a given environment or plant host.

Leaf spot of eucalyptus is a disease complex caused by numerous species of fungi of the genus *Teratosphaeria* (Crous et al., 2009). Diseased eucalyptus plants sampled from South Africa yielded two fungal species (i.e., *Teratosphaeria juvenalis* and *T. verrucosa*) that co-occur in the same leaves and even in the same spots. In Australia, *T. gauchensis* and *T. zuluensis*, (which predominantly cause eucalyptus stem cankers) have been reported to occur in leaf spots either alone or in association with some of the other species belonging to *Teratosphaeria* (Crous et al., 2009). On mango, a complex disease known as mango malformation is caused by *F. mangiferae, F. mexicanum, F. proliferatum, F. pseudocircinatum, F. sterilihyphosum,* and *F. tupiense* where individual species of this fungus prevail in association with the symptomatic tissues (Freeman et al., 2014).

The importance of the temporal order of host infection by different pathogens is another important factor to be considered. Several reports have been described in the literature in this regard. Le May et al. (2009) demonstrated that the simultaneous inoculation of two plant pathogenic fungi (*Mycosphaerella pinodes* and *Phoma medicaginis* var. *pinodella*) associated to the *Ascochyta* blight disease complex limits disease development and their reproduction. However, when plants pre-inoculated with one pathogen were then inoculated with another there was a marked increase in severity of the disease. In contrast to this report, Sagar and Sugha (1997) reported a decrease in necrotic symptoms of pea caused by *Phoma medicaginis* var. *pinodella* previously inoculated with the vascular root pathogen *F. oxysporum* f. sp. *pisi*. The order/succession of host infection by each pathogen in a complex disease and its trophic level might affect the nature of interaction that these pathogens eventually develop during disease occurrence. Such temporal effects could also be related to the notion of ecological niche.

There are also reports of synergistic interactions between more or less aggressive strains of numerous pathogens. Kaur et al. (2011) for example demonstrated that the severity of white rust symptoms (caused by the pathogen *Albugo candida*) on mustard increases and the symptoms appear earlier when a host highly susceptible to *A. candida* but resistant to *Hyaloperonospora parasitica* (the causal agent of downy mildew), was first inoculated on day 1 with a less aggressive strain of *H. parasitica* followed by a 10-days-post inoculation with an aggressive strain of *A. candida*. Taken together, the outcomes due to co-occurrence of the same pathogens on the same host may result in antagonism and/or synergism which are likely influenced by the order of their association with the infected plant. In-depth future studies are needed to consider the temporal aspect of infection by different pathogens and their interactions, both among themselves and with the plant, to uncover the possible underlying mechanisms.

### Virus–Virus Interactions

Disease synergisms among two or more plant pathogenic viruses increasing the severity of symptoms has been reported on a variety of crop species (**Table 1**). For example, *Maize chlorotic mottle virus* (MCMV) and *Wheat streak mosaic virus* (WSMV) cause corn lethal necrosis. The interactions between these two viruses results in a significant increase (up to 10-fold) of the MCMV concentration in plants (Scheets, 1998). In addition, WSMV infection is considerably enhanced by the presence of MCMV both in terms of frequency and intensity. Likewise, a strong synergistic interaction was found between *Cucumber mosaic virus* (CMV) and black-eye *Cowpea mosaic virus* (BCMV) in severely stunted cowpea in fields (Pio-Ribeiro et al., 1978). In an experimental inoculation study, each virus caused relatively mild disease when inoculated singly and plants showed significantly reduced stunting. In contrast, disease severity and the extent of stunting increased when these two viruses are co-inoculated under the same conditions. Another example is blackberry yellow vein disease (BYVD) complex which is caused by the cooperation between different viral species (Martin et al., 2013). More specifically, *Tobacco ringspot virus*, *Raspberry bush dwarf virus,* and a new virus which belongs to the genus *Crinivirus* are involved. The BYVD disease severity was stringently related with the number of viruses infecting plants. No disease symptom was caused by the incoming viruses in single infections while symptoms clearly become visible in mixed infections. Hence, in some cases the absolute number of viruses infecting plants is likely to be more important than the type of viruses involved in the infection. However, the role of vectors in transmitting complex viral diseases is not clear in the above described examples. For example, are the viruses commonly transmitted by the same vector? If this is the case, are there likely to be differences in the acquisition time or latency of the organisms that could affect whether they are co-transmitted? Answers to these questions are likely to be important in disease control; however, information available to date is not sufficient and further studies are required.

In addition to synergisms between plant viruses, several studies reported mixed virus infections leading to mutual exclusion (antagonism); this aspect has been recently reviewed by Syller (2012).

### Mixed Interactions

There are a few reports in the literature of plant disease complexes involving association of more than one pathogenic microbial phyla (**Table 1**). An example is brown apical necrosis of walnut fruit where numerous plant pathogenic fungi (*Fusarium, Alternaria, Cladosporium, Colletotrichum,* and *Phomopsis*) and a bacterium (*Xanthomonas arboricola*) are involved (Belisario et al., 2002). Another example is root rot disease complex of *Panax notoginseng* where a large number of plant pathogenic fungi (*Alternaria panax, Alternaria tenuis, Cylindrocarpon destructans, Cylindrocarpon didynum, F. solani, F. oxysporum, Phytophthora cactorum, Phoma herbarum,* and *Rhizoctonia solani*) and bacteria (*Pseudomonas* sp. and *Ralstonia* sp.) have been found (Miao et al., 2006; Ma et al., 2013). The mechanisms of interaction that result in communication and synergism of pathogens in these complex diseases are currently unknown.

## New Approaches are Needed for Studies of Complex Plant Diseases

An initial thorough analysis that correctly identifies the disease causing agent(s) is the primary step of managing a plant disease (Adams et al., 2013). Suitable disease management tools can then be applied such as the use of an anti-microbial compounds which can be administered depending on the plant type and part affected by the disease. Although the application of a chemical substance can be of importance, a more sustainable disease management can be achieved only through the development of more long-term strategies. To this aim, a better knowledge of pathogen–pathogen synergism in causing complex plant diseases is of paramount importance. For example, many anti-microbial strategies currently used in agriculture are specific to control a given microbial pathogen. Targeted chemical control strategies become limiting when more than one pathogenic agent contributes to the disease as the application of the specific substance may not necessarily result in successful disease management. It is therefore important to study plant disease complexes, the synergisms in pathogen–pathogen interactions as well as the underlying mechanisms to identify important links that may be manipulated to ensure crop health. This could be a difficult task since disease complexes are related to environmental conditions, cultural practices, and geography (Willocquet et al., 2002). It is therefore important to design the experimental approach leading to identification of pathogen cortege in relation to the crop production system.

The diagnosis and management of complex diseases can be lengthy resulting in significant yield losses. Often the use of classical isolation techniques on selected or semi-selected media may not yield any of the causal agents or sometimes only their partial isolation. Because of the complexity of polymicrobial diseases, the study in this regard was somewhat overlooked in the past. In most of the studies reported in **Table 1**, the authors performed the isolation of pathogen on culture growth media. In addition, other more specific (e.g., immunofluorescence or PCR) or generic (e.g., morphological identification) assays were used. However, currently we have new knowledge and techniques which may facilitate the understanding of the total microbial species involved in plant diseases as well as the underlying mechanisms. Hence, studies of complex diseases now need to benefit from culture-independent analyses (high-throughput sequencing for example). This approach does not have the limitations of the classical culture-based approach, which is often lengthy and costly (Nikolaki and Tsiamis, 2013). In the modern era of biodiversity surveillance, techniques such as next-generation sequencing (NGS) have enabled high-throughput analyses of complex microbial populations (reviewed by van Dijk et al., 2014). This has transformed microbiology and has revealed that microbial diversity is vastly underestimated based on classical cultivation-based techniques (Gilbert and Dupont, 2011). In the last 10 years, metagenomic projects have been combined with NGS technologies boosting studies in microbial ecology at a very fast pace (Venter et al., 2004; Tringe and Hugenholtz, 2008).

Although plant pathology in general and plant disease complex in particular could stand to gain from exploiting NGS and metagenomic approaches, the current literature reveals only a limited number of applications of this technology. Only a few studies have applied such methods with a regard to the diagnosis of new pathogens (Adams et al., 2009, 2013). For example, sequences of an entire viral genome (determined via a single step of high-throughput parallel sequencing) highlighted the presence of three novel viruses in sweet potato plants which were infected with known pathogenic viruses occurring at extremely low titers (Kreuze et al., 2009). These novel technologies are thus a powerful tool to understand the implication of two or more microbes and their contribution in plant disease occurrence.

There are some limitations in using these novel OMICs methods with respect to studying the role of microbial consortia and plant disease. Although these will help to better characterize complex diseases they will not necessarily allow to determine which microbe is the dominant factor in the disease occurring process. Identification of the infection-site derived nucleic acid sequence is not unequivocal evidence that the microorganism in question is the causal agent of disease. For example, Adams et al. (2009) determined the complete virus genome sequence of a pathogenic virus via metagenomic anaylsis. This data is convincing proof that a transmissible infection with virus-like symptoms was linked with the occurrence of the new *Cucumovirus* full genome present in the infection site despite the fact that Koch’s postulates were not fulfilled. Importantly, viral particles were not observed nor the disease was re-established in the original host highlighting that only the virus presence can be determined via metagenomic sequence analysis. Metagenomics will therefore pose a new challenge for taxonomy and role of phytopathogens in disease (Studholme et al., 2011). Another aspect when considering mixed infections will be that of distinguishing pathogens from saprotrophic microorganisms.

Another important feature in studying complex diseases concerns growth models and the pathogen cortege. Examples are RICEPEST (Willocquet et al., 2002) and WHEATPEST (Willocquet et al., 2008) models which have been developed to simulate yield losses due to several pests under different production situations. These models are the first to include the impact of several diseases on yield losses. A limit to these models is that although impact of the different diseases is considered, they do not take into account the potential interaction between the pathogens. Future studies need to take into account such limits in order to develop models that simulate the possible synergism between plant pathogens.

Recently, Elena et al. (2014) proposed that evolutionary game theory provides an adequate theoretical framework to analyze mixed viral infections and to predict the long-term evolution of the mixed populations. Here we recommend that the same approach can be used to analyze mixed fungal and bacterial infections which need to be considered in the future studies.

## What Mechanisms are Known?

Synergistic interactions between pathogens in humans have been reported to occur through several mechanisms such as chemical signaling influencing gene expression or via metabolic exchange/complementarity in order to avoid competition for nutrients and improve metabolic ability of the consortium (Frey-Klett et al., 2011). Pathogen–pathogen interactions are also known to result in viral induced bacterial adhesion, interference with the host immune system, production of viral products, direct bacterial effectors and viral-derived disruption of the epithelium (Singer, 2010; Peters et al., 2012; Bosch et al., 2013). On the other hand, studies involving mixed infections in plant diseases are still in their infancy and the underlying mechanisms of possible synergistic interactions are currently unknown. However, it has been recently reported that a plant pathogen undergoes interspecies signaling via quorum sensing signals with residential commensal microbiota indicating the occurrence of intimate multispecies interactions *in planta* (Hosni et al., 2011). Signaling among different bacterial species is also likely to play an important role in the synchronization of behaviors as well as expression of virulence factors in mixed populations. The example of Hosni et al. (2011) could pave the way for the discovery of other interspecies interactions among microbes living in close association with plants.

Synergistic interactions among bacterial pathogens could also be indirect as for example the ability of certain harmless or beneficial plant-associated bacteria and pathogens to suppress host immunity or alter the plant micro-environment that can promote colonization by other pathogens. Several studies have demonstrated that plant defenses are induced following the infection of an avirulent bacterial strain resulting in its own growth restriction as well as that of a co-inoculated virulent strain (Klement and Lovrekovich, 1961; Averre and Kelman, 1964; Omer and Wood, 1969). The growth of non-pathogenic bacterial strains could reach higher cell numbers when co-inoculated with pathogenic bacteria (Young, 1974).

On the other hand, growth differences of virulent bacterial species could occur when they are co-inoculated owing to variations in fitness rather than virulence (Llama-Palacios et al., 2002). Similarly, growth interference in a mixed infections between different strains of *P. syringae* has been reported to be influenced by the initial population size (Macho et al., 2007). The lowest range of initial population necessary to circumvent interference has been reported to be dependent both on the type of virulence factor that differentiates the co-inoculated strains as well as the pathogen aggressiveness. In another study, growth interference in a mixed infection between different strains of *P. syringae* is strain-dependent and the populations of strains in mixed inoculations were lower than those in independent inoculations (Bartoli et al., 2015). The mechanisms supporting these synergistic and/or antagonistic interactions need to be explored taking into account the importance of immune suppression or modulation of phytohormone-based signaling mechanisms.

The mechanism of synergistic interaction among the *Sweet potato feathery mottle virus* and *Sweet potato chlorotic stunt virus* has been investigated and shown to be mediated by the SPCSV encoded RNase3 protein (Cuellar et al., 2009). The authors suggest that RNase3 may synergize *Sweet potato feathery mottle virus* and other viruses by targeting a specific host component via interference with small-RNA biogenesis; the precise mechanism of this interaction is currently unknown.

A previous study (García-Marcos et al., 2009) on synergistic interactions between *Potato virus X* and *Potato virus Y*, which led to an increased systemic infection in *Nicotiana benthamiana*, reported transcriptional changes and oxidative stress associated with the synergistic infection. This stress correlated with the misregulation of antioxidative genes in a microarray experiment. Expression of genes encoding oxylipin biosynthesis were upregulated by the synergistic infection caused by the two viruses and were not by single infection with *Potato virus X* or *Potato virus Y.* Interestingly, oxylipin biosynthesis genes were recently shown to positively regulate programmed cell death during compatible infections with the synergistic pair *Potato virus X-Potato virus Y* and *Tomato spotted wilt virus* (García-Marcos et al., 2013).

## Polymicrobial Diseases and Koch’s Postulates

Of the four criteria postulated by Koch [they are (i) the microbe must be isolated from an infected host and obtained in pure culture; (ii) it must cause infection when inoculated into a healthy host; (iii) it must be re-isolated from the inoculated, symptomatic organism; and (iv) it must be shown to be the same as the originally cultured microorganism], some are not valid for polymicrobial infections as for example re-inoculation of the pathogen not necessarily causes disease if synergism is lacking. Another postulate raised by Koch was that the pathogen should be isolated from diseased and not from healthy organisms. This is also challenged for an increasing number of human diseases as well as plant diseases. Pierce’s disease of grapevine is an example, where the bacterial pathogen *Xylella fastidiosa* can colonize xylem vessels of asymptomatic plants for a long period of time (Purcell, 2013). Another issue related to these postulates are the non-culturable pathogens (Oliver, 2010) for which none of the Koch’s criteria can be fulfilled. The identification of polymicrobial diseases and other issues, some raised here, suggests that Koch’s postulates need to be revised. For example, pathosystems may be divided into two groups; simple diseases in which only one organism is involved, and complex diseases in which there is an interplay of more than one organism (e. g., insect, commensal, pathogen, non-culturable organism etc.). For the complex diseases, an additional criterion (fifth) taking into account the interactions between microrganisms involved in disease occurrence that are positively correlated with disease occurrence and/or severity might be necessary.

## Concluding Remarks

The pathogenic microorganisms associated with plants now need to be isolated/studied with the view that possible antagonistic, mutualistic, or synergistic interactions are taking place. A careful assessment of the roles of all the microorganisms isolated from the infection sites needs to be evaluated as multispecies interactions and consortia can be involved in establishment and aggravation of the disease. This will need to involve the interdisciplinary research collaboration between bacteriologists, mycologists and virologists. Understanding the biology and molecular interactions of these inter-microbial processes may be important in defining new targets and strategies for disease control.

### Conflict of Interest Statement

The authors declare that the research was conducted in the absence of any commercial or financial relationships that could be construed as a potential conflict of interest.
